# Health system plan for implementation of Paris agreement on climate change (COP 21): a qualitative study in Iran

**DOI:** 10.1186/s12889-020-09503-w

**Published:** 2020-09-11

**Authors:** Arefeh Mousavi, Ali Ardalan, Amirhossein Takian, Abbas Ostadtaghizadeh, Kazem Naddafi, Alireza Massah Bavani

**Affiliations:** 1 Department of Health in Emergencies and Disasters, School of Public Health, Tehran University of Medical Sciences, Tehran, Iran; 2 Department of Climate Change and Health, Institute for Environmental Research, Tehran University of Medical Sciences, Tehran, Iran; 3 Department of Global Health & Public Policy, School of Public Health, Tehran University of Medical Sciences, Poorsina Ave, Tehran, Iran; 4 Department of Management and Health Economics, School of Public Health, Tehran University of Medical Sciences, Tehran, Iran; 5 Health Equity Research Center (HERC), Tehran University of Medical Sciences, Tehran, Iran; 6 Center for Water Quality Research, Institute for Environmental Research, Tehran University of Medical Science, Tehran, Iran; 7 Department of Environmental Health Engineering, School of Public Health, Tehran University of Medical Sciences, Tehran, Iran; 8 Department of Irrigation and Drainage Engineering, College of Abureyhan, University of Tehran, Tehran, Iran

**Keywords:** Climate Change, Paris agreement, Mitigation, Adaptation, Health system, Iran

## Abstract

**Background:**

Ensuring public health is crucial in any policy debate on climate change. Paris Agreement on climate change is a global contract, through which countries have committed themselves to a public health treaty. The agreement has laid the foundation for mitigation and adaptation. This study was conducted to provide an evidence-based framework for policy-making in the health system of Iran in order to reduce the adverse effects of climate change on public health and to increase the adaptation of the health system as a result.

**Methods:**

This is a qualitative study. We first used Delphi method to extract the components of Paris Agreement on climate change that were related to the functions and policymaking of health system in Iran. Twenty-three experts in health and climate change were identified purposefully and through snowball sampling as participants in Delphi. Data collection instrument was a structured questionnaire. We used SPSS software version 25 for data analysis based on the descriptive indices including the mean, the percentage of consensus above 75%, and the Kendall coordination coefficient.

**Results:**

Seventy-nine components classified within nine categories were extracted. The most important examples of the implementation of Paris Agreement on climate change in the health system of Iran were: participation in the formulation of strategies for mitigation and adaptation, identifying vulnerable groups, assessing vulnerability, increasing the capacity of health services delivery during extreme events, using early warning systems, using new technologies to increase the adaptation, evaluation of interventions, financial support, increasing the number of researches, increasing the knowledge and skills of staff, and finally public awareness.

**Conclusions:**

Evidence-based policy-making is pivotal to develop effective programs to control the health effects of climate change. This research provided policy translation and customization of micro and macro provisions of Paris Agreement on climate change, in line with the political context of health system in Iran. Our finding will pave the ground, we envisage, for further steps towards capacity building and enhancement of resiliency of the health system, adaptation interventions, and evaluation, identification of barriers and facilitators for adaptation and decreasing the adverse health effects caused by the climate change, in Iran and perhaps beyond.

## Background

Climate change is one of the major challenges of human beings in the twenty-first century [1], and a big threat to the achievement of Sustainable Development Goals (SDGs) [2]. Given the dramatic consequences of climate change and its effect on the economic, social, and environmental structure of human societies, “climate action” has been identified as the 13th goal of the SDGs to combat the climate change and its consequences [3]. Climate change and its health effects are beyond the national boundaries. All the possible consequences of the climate change such as heat waves, storms, forest fire, floods, or droughts directly threaten the health and life of human beings. Climate change also affects public health indirectly through the ecosystems, agriculture, changes in pathogens’ life cycle, plus the quantity and quality of water, food, and air. Worse still, some consequences of climate change, e.g. migration, conflict, and controversy over limited resources, may influence the social determinants of health (SDH) [4, 5].

Ensuring public health concerns is a crucial consideration in any policy debate on climate change. The World Health Organization (WHO) and the Secretariat of the United Nations Framework Convention on Climate Change (UNFCCC) strongly pursue the policies to tackle the adverse effects of climate change. In the same vein, at the First Global Conference on Health and Climate in August 2014 in Geneva, 360 participants from various communities and governments discussed all aspects of climate change and health [6]. Also, the conventions of the World Health Assembly (WHA) treat the health effects of climate change as a global subject, and consider combating the consequences of climate change as a joint problem, highlighting the responsibility of all member states to combat climate change [7].

So-called COP 21, Paris Agreement on climate change is a global commitment signed by representatives of most countries from around the world, which began a new era in a global response to serious threat caused by climate change. The treaty asks countries to take certain sets of action to reducing the carbon emissions and limiting the global warming to a maximum of 2 °C until the end of the twenty-first century [8]. At present, the world has a climate treaty, by which countries also commit to the public health treaty implicitly [9]. As set out in the agreement, the “right to health” is the focal point for countries in this regard [10]. The implementation of COP 21 can provide a phenomenal opportunity for WHO to build a healthier society [11]. Based on Paris Agreement, almost no country has provided the policies and investment to decrease the effects of climate change on SDH [12]. If the current processes of economic activities are pursued regardless of the Paris Agreement, the world will face with unfair distribution of power, wealth, and health, and poor health continues to harm those having less income and wealth. Therefore, interventions in public health and climate change are needed to create healthier and more balanced communities [12].

In line with the global trend, Iran as an upper-middle-income economy [13], Located in West Asia, with over 82 million population in 2017 [14], is also heavily influenced by climate change and its various consequences, including its adverse effects on the health. In addition, the Human Development Index (HDI) was 68 in 2014 [15]. Some other indicators are shown in Table 1.

**Table 1:** Some health indicators, demographic and economic estimates among Iranian population [16–19]

Indicators		Estimate rate
**Demographic**	Population living in urban areas (2013)	72.3%
	Population growth rate (2017) [12]	1.1%
	Population aged 65 or older (2015) [13]	4.9%
	Population under five (2015) [13]	8.8%
**Health**	Life expectancy at birth m/f (2017) [18]	75.5/79.4 years
	Under-5 mortality per 1000 live births (2018) [19]	14
**Economic**	GDP per capita (current US$, 2017) [18]	17,519 USD
	Total expenditure on health as % of GDP (2014) [19]	6.9%

Because of improved health standards, the establishment of national surveillance systems, changing lifestyle and increasing urbanization rates, the pattern of diseases has been transitioning from communicable diseases towards non-communicable diseases (NCDs) in the course of last two decades [20]. Previous studies showed that air pollution and other chemical contaminations have increased the burden of non- communicable diseases (NCDs), e.g. cardiovascular disease (CVDs), respiratory diseases, and cancer in Iran [21]. NCDs accounted for 82% of all deaths in 2018 in Iran [22]. A study conducted in Tehran demonstrated the CVD mortality burden is associated with air pollution [23]. It is estimated that 79,200 and 9100 deaths per year are the results of environmental factors and potential outcomes of outdoor air pollution respectively [24]. In terms of communicable diseases, with the burden of 9.7% in 2012 [21], Malaria, leishmaniasis, Crimean-Congo Hemorrhagic Fever (CCHF), diarrhea, and cholera were the most important infectious diseases associated with the climate change in the course of last few decades in Iran [25]. Given the growing influences of diseases associated with climate change as well as the growing trend of climate disasters in Iran, the need for evidence-based health policies to combat the consequences of climate change is now more than ever.

The Ministry of Health and Medical Education (MoHME) is responsible for providing health services through at all levels including Primary healthcare network (PHC) and hospital care. Since 2014, so-called Health Transformation Plan (HTP), a series of reforms have been conducted in the health system with the aim of reaching Universal Health Coverage (UHC) [26]. The MoHME is also responsible to deal with the medical aspects of environmental hazards, especially climate change, disaster management, environmental and occupational health. These centers work closely with the Department of Environment (DoE), and the Institute for Environmental Research (IER). Indeed, The MoHME, DoE and IER are the three organizations responsible for policymaking and managing the health impacts of climate change [27]. Despite all the efforts made in this field, Iran’s health system has been facing some challenges in dealing with the consequences of climate change, i.e. the weak collaboration among related stakeholders, insufficient technical, organizational, financial and human resources capacities, inadequate high-quality research as well as some financial shortcoming [27].

As the steward of health system, MoHME is committed to implement the WHA’s treaty 2008 to protect population health against climate change in Iran. This is a complex and multi-dimensional task, fulfilling which requires intersectoral collaboration to enhance the awareness of multiple stakeholders and their adaptation. To do so, the MoHME, through its leadership and coordination capacity to improve population health, is expected to develop an effective and comprehensive framework that addresses all issues related to the mitigation and adaptation to the adverse health effects of climate change. This study aimed to extract the components of functions and policymaking to provide an evidence-based framework for health policy-making towards reducing the GHG emissions and adapting to the health effects of climate change in Iran.

## Methods

This is a qualitative study. First, we used Delphi method to extract the components of Paris Agreement on climate change that were related to the functions and policymaking of health system in Iran. Delphi is a systematic method to receive the opinions of a group of experts in analyzing a topic and discussing the complex issues [28, 29]. This approach leads to the conversion of different views and opinions into one or more common concepts through an iterative feedback process [30]. The experts provided their opinions on the same questions at least twice, while the negative influences of personality and the status of the participants in the responses of others are prevented [31, 32].

### Study instruments and data collection

We developed a structured questionnaire in the form of open/closed -ended questions for data collection. To develop the questionnaire, we first read the draft of Paris Agreement on climate change. The agreement includes 29 articles addressing the various issues regarding the requirements of countries on two pillars of “mitigation” and “adaptation” to the climate change phenomenon. Considering the comprehensiveness of the provisions outlined in the agreement, we identified all related and unrelated components of health and entered them into an Excel file. A structured questionnaire consisting of 47 items was designed and experts were asked to identify the health-related components amongst all components extracted from the draft of agreement within a five-point Likert scale. These components were identified as health-related components that directly or indirectly affect public health measures. In all three Delphi phases, participants’ consensus rate was determined by a five-point Likert scale. Thus, the number five indicated the highest level of agreement rate, and the number one showed the lowest level of the agreement upon the components. To ensure the content validity and face validity in all rounds of the Delphi, three external experts independent of the Delphi panel have been selected to provide some assurance of validity. After the return of questionnaires in all rounds of Delphi, the external investigators reviewed content to approve a list of health components of the Paris Agreement identified by the expert panel. This process provides some assurance of content validity.

During the first round of Delphi, we provided the questionnaire along with the relevant instructions to the experts, both physically and through email. Upon receiving the first round, we then formulated these components again in the second round and in the form of a questionnaire with open questions. We used an electronic questionnaire design software (called Cafepardazesh, The URL address: http://www.cafepardazesh.ir/form/view.php?id=14363339), and provided the respondents with its link. We asked participants to express policies, specific strategies, and action plans of Iran’s health system for implementing the components of the first round. We then reviewed the views and deleted repetitive and similar items. Given the diversity and complexity of the subject presented in this round, and for better understanding and interpretation of results, the components obtained from the second round were once again set up in a structured questionnaire within 9 categories and on a five-point Likert scale, which were again confirmed by our research team and were sent to the experts for the third round of Delphi to reach consensus. This classification was made by qualitative codes obtained from the previous round. In this round, the validity and reliability of the questionnaire were examined through qualitative methods. Structured processes were therefore used to record, write, and interpret the data. In addition, the findings, interpretations, and results of the research were again reviewed by a group of experts.

### Sampling

Through snowball approach, we used purposeful sampling to identify 23 faculty members, researchers, and experts with scientific and practical qualifications in the field of health and climate ​​change (see Table 2). To enhance the generalizability of findings, we also sought the opinions of the executive directors of the health system with at least 2 years’ experience in the field of climate change. All participants were aware of the research objectives and their consent to participate in the study was obtained. The entire Delphi process was performed anonymously.

**Table 2:** Participants’ profile in Delphi process

	First round	Second round	Third round
Response rate	(20/23) 86%	(20/20) 100%	(16/20) 80%
**Specialty (Number of people)**			
Environmental health	6	6	4
Health in disasters and emergencies	2	2	2
Non-communicable diseases	3	3	2
Communicable diseases	2	2	2
Climate change and health	4	4	3
Nutrition	2	2	2
Medicine	1	1	1
**Sectors**			
Academic	9	9	7
Executive	11	11	9

### Data analysis

Data analysis was performed using SPSS software v. 25. The analysis of the responses of the first round was performed based on the descriptive indices including the mean and the percentage of consensus. The analysis of the agreement and disagreement among the experts was based on the five-point Likert scale. Considering the sensitivity of the subject, the components with the score of 3 and above were selected as health-related criteria in the first round. The analysis of second-round responses was performed based on qualitative codes, and the components were classified within 9 categories. In the third round, the average and the percentage of the consensus of experts were calculated, and the components with the score of 4 and higher were included in the study. This round also allowed the experts to receive controlled feedback. Therefore, the experts reviewed their opinions and omitted, interpreted, corrected, and evaluated the peers’ opinions. In all rounds, the consensus above 75% was included [33].

**Fig. 1:**
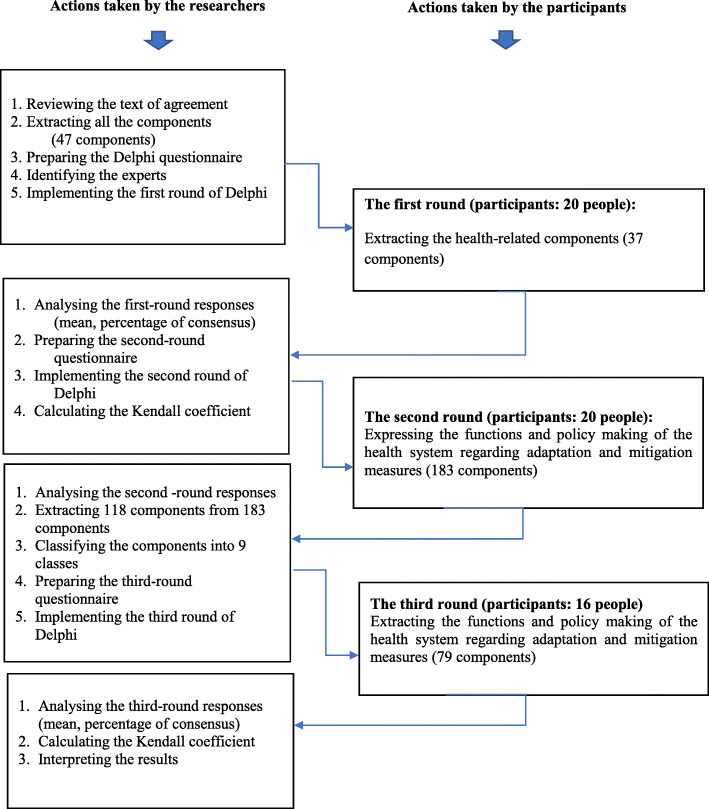
Summary of the methods and measures taken in the study

Although we reached data saturation after the third round, to decide whether to stop or continue Delphi rounds, the Kendall coordination coefficient was calculated. This scale can be used to determine the validity of the opinions of the referees and the degree of consensus among the experts [34]. The value of this scale is 1 in case of complete consensus, and is 0 in case of absolute disagreement. An unchanged or slightly increased coefficient in two consequent rounds shows that no increase has been occurred in the consensus and the opinion polling process should be stopped [34, 35]. Figure 1 shows a summary of the study methodology and fundamental steps taken.

## Results

### Components of Paris agreement on climate change

By carefully reviewing the text of the Paris Agreement on climate change and polling the expert opinions in this field, 47 components related and unrelated to health were extracted. The components were classified according to the main pillars of the agreement, namely “mitigation” and “adaptation”.

### Results of the first round of Delphi

Out of 23 distributed questionnaires, 20 were returned, (the response rate of 86%). Out of 47 components identified in the previous step, 37 health-related components were identified in the Paris Agreement on climate change. The mean and percentage of consensus among the experts in each of the components is reported in Table 3.

**Table 3:** The consensus over 75% on health-related components in Paris Agreement on climate change

	Health-related components of Paris Agreement on climate change	Mean ± SD	Consensus (%)
1	Developing strategies, plans and implementing the mitigation measures	3.65 ± 1.30	75%
2	Increasing the ability and capacity on local, national, regional, and international levels to adapt to the adverse effects of climate change	4.10 ± 1.11	90%
3	Creating the adaptation based on the current, past, indigenous, and local knowledge	3.65 ± 1.13	85%
4	Integrating the adaptation in the policies, social, economic, and environmental measures	4.10 ± 0.96	90%
5	Creating country-oriented, gender-oriented, and participatory adaptation with regard to sensitive groups, communities, and ecosystems	3.15 ± 0.99	85%
6	Adjustment, implementation and monitoring of National Adaptation Strategies and Plan of Actions	4.40 ± 0.88	95%
7	Capacity building for mitigation measures based on the local, national, and regional needs	3.35 ± 1.18	75%
8	Capacity building for adaptation measures based on local, national, and regional needs	3.90 ± 1.02	90%
9	Improving the resiliency to climate change	4.15 ± 0.93	90%
10	Reducing the vulnerability to climate change	4.30 ± 0.92	90%
11	Promoting voluntary cooperation in climate change measures	3.75 ± 1.01	90%
12	Encouraging and facilitating the participation of public and private institutions in climate change measures	3.85 ± 1.13	80%
13	Developing and transferring the technologies to increase the adaptation	3.70 ± 1.12	85%
14	Providing financial support for developing and transferring the technologies at different stages of the technology life cycle	3.60 ± 1.42	75%
15	Providing financial resources to increase the adaptation and resilience to climate change	3.45 ± 1.43	90%
16	Assessing the adequacy and effectiveness of adaptation measures	3.65 ± 1.22	80%
17	A systematic monitoring of climate system and early warning systems	3.70 ± 1.30	85%
18	Assessing the effects of climate change	4.15 ± 1.13	90%
19	Assessing the vulnerability to climate change	4.10 ± 0.96	90%
20	Minimizing the damage caused by the effects of extreme climate events	3.60 ± 1.35	80%
21	Developing early warning systems for extreme climate events	3.45 ± 1.27	80%
22	Being prepared for extreme climate events	4.00 ± 0.97	95%
23	Assessing the damages caused by the extreme climate events	3.65 ± 0.87	90%
24	Risk management of extreme climate events	3.90 ± 0.96	90%
25	Comprehensive risk assessment of extreme climate events	3.30 ± 1.38	70%
26	Strengthening the scientific knowledge and research about climate change and its effects	4.35 ± 0.93	95%
27	Promoting the education on climate change and its effects	4.40 ± 0.75	100%
28	Promoting the research on climate change and its effects	4.30 ± 0.73	100%
29	Integrating and sharing of information, knowledge, appropriate actions, experiences, and lessons learned	4.05 ± 0.75	100%
30	Providing timely and accurate reporting of information	3.75 ± 1.37	85%
31	Using lessons learned from the International Climate Change Convention frequently and effectively	4.05 ± 1.05	95%
32	Providing regular reports on the progress of the policies, programs, and undertaken actions	3.95 ± 1.05	95%
33	Raising the public awareness on climate change and its effects	3.30 ± 0.97	90%
34	Raising the public awareness and transparency on mitigation measures	3.80 ± 1.05	85%
35	Raising the public awareness and transparency on increasing the adaptation and resiliency measures	4.30 ± 0.92	95%
36	Promoting the public participation on measures related to climate change	4.15 ± 0.87	95%
37	Promoting the public access to information on climate change and its effects	4.05 ± 0.99	90%

### Results of the second round

In this round, out of 20 questionnaires that were distributed, 20 questionnaires were returned (the response rate of 100%). In this round, regarding that for each of the 37 components related to health in phase one, several strategies and action plans were presented in the second phase, 183 items were introduced according to the experts’ opinions. After integrating the similar items, 118 components related to the functions and policymaking of health system in Iran were extracted from Paris Agreement on climate change. These components were classified into nine categories including mitigation, capacity building, advocacy, technology development and transfer, financial affairs, assessment, evaluation and monitoring, education and research, reporting and sharing information, and components for public.

### Results of the third round

Out of 20 questionnaires distributed during the third round, 16 questionnaires were returned (the response rate of 80%). Out of 118 components related to the functions and policymaking of health system in Iran, experts agreed upon 79 components. The mean and percentage of consensus on each of the components is reported in Table 4. For the second and third rounds, the Kendall test was found to be significant (*P*.value: 0.000), and the coefficients of 0.217 and 0.239, were obtained respectively. The Kendall coefficient increased by 0.022 in two consequent rounds. This small difference in the Kendall coefficient reflects the consensus and polling stop.

**Table 4:** The consensus over 75% on components related to the functions and policymaking of health system in Iran extracted from Paris Agreement on climate change

	**Pillar1: Mitigation**	**Mean ± SD**	**Consensus (%)**
1	Limiting the greenhouse gas emissions from health buildings and facilities	4.00 ± 0.73	75%
2	Participation of the Ministry of Health in formulation of strategies, programs and implementation of mitigation measures by emphasizing on the health effects of climate change	4.37 ± 0.80	93.8%
3	Improving the efficiency of energy carriers and using renewable and clean energies in health centers and all health-related manufacturing units, including cosmetics, health, and pharmaceuticals	4.12 ± 0.50	93.8%
4	Reducing the energy, water, and waste production consumption in the health services and manufacturing centers	4.18 ± 0.54	93.8%
5	Reducing fossil fuel consumption in the health services and manufacturing centers	3.93 ± 0.92	81.2%
6	Intergovernmental cooperation in formulation and implementation of mitigation laws	4.31 ± 1.07	87.5%
7	Paying attention to the resolutions of the supreme council regarding health and food security on formulation of laws on climate change	4.25 ± 0.68	87.5%
Pillar 2: Adaptation			
	**Capacity building**	**Mean**	**Consensus (%)**
8	Increasing the capacity of healthcare infrastructures and providing safe facilities based on population needs and density for more coverage of health services related to climate change consequences	4.18 ± 0.65	87.5%
9	Implementing structural, non-structural, and functional measures in hospitals and health centers to reduce the vulnerability to the consequences of extreme climate events	4.00 ± 0.89	75%
10	Using the laboratory networks to quickly detect and control the communicable diseases sensitive to climate change	4.12 ± 0.95	75%
11	Increasing the access to safe and sanitary water	4.87 ± 1.25	75%
12	Using indigenous and local capacity for adaptation to health effects of climate change	4.18 ± 0.83	87.5%
13	Developing the guidelines for protecting and adapting the people in jobs sensitive to climatic variables	3.93 ± 1.18	75%
14	Developing the strategies for resilient health system to cope with the health consequences of climate change	4.00 ± 1.09	87.5%
15	Promoting the healthcare procedures in order to cope with the adverse health effects of climate change	4.18 ± 0.75	81.2%
16	Identifying the vulnerable groups in relation to the consequences of climate change and performing special protective interventions for them	4.43 ± 0.89	87.5%
17	Participation of the health system in formulation of national adaptation plan	4.62 ± 0.61	93.8%
18	Developing the public health insurance coverage to increase the adaptation to the consequences of climate change	4.18 ± 0.75	81.2%
19	Formulating the protocols for identification of the diseases and healthcare intervention for diseases sensitive to climate change	4.62 ± 0.80	93.8%
20	Establishing the membership of the Ministry of Health in national and international working groups on climate change	4.56 ± 0.51	100%
21	Applying prevention and control measures for emerging and re-emerging diseases caused by climate change	4.62 ± 0.55	100%
22	Applying the interventions to control climatic risk factors	4.43 ± 0.62	93.8%
23	Providing the provincial adaptation plans according to their risks	4.56 ± 0.62	93.8%
24	Providing the health services related to the health effects of climate change equitably and without discrimination on the grounds of sex and ethnicity	4.25 ± 1.06	87.5%
25	Providing the national plans for nutrition management in extreme climate events such as floods, heat waves, and cold waves	4.18 ± 0.65	87.5%
26	Providing the National Adaptation Strategy and Plan of Action (NASPA) for health issues related to climate changes by the Ministry of Health	4.81 ± 0.40	100%
27	Providing the healthcare preparedness plans for extreme climate events to reduce the mortalities and injuries	4.43 ± 0.51	100%
28	Creating an Incident Command System (ICS) in case of extreme climate events to quickly respond to such emergencies	4.25 ± 0.68	87.5%
29	Saving health-related resources in order to become prepared against the extreme climate events	4.06 ± 0.68	81.2%
30	Integrating the comprehensive risk assessment plans for extreme climate events in the health service delivery system	4.31 ± 0.60	93.8%
	**Advocacy**	**Mean**	**Consensus (%)**
31	Involving the participation of the public and private institutions in the measures related to climate change	4.00 ± 0.89	75%
32	Performing the intergovernmental cooperation on nutrition policies, especially in critical situations	4.00 ± 0.96	81.2%
33	Implementing the international agreements and partnerships to control the communicable diseases through the borders	4.56 ± 0.72	87.5%
	**Technology development and transfer**	**Mean**	**Consensus (%)**
34	Collaboration of the Ministry of Health with the universities in applying new technologies for adaptation	4.06 ± 0.77	75%
35	Reducing the waste production in the health centers and hospitals using modern methods of re-engineering the processes and upgrading the existing systems	4.23 ± 0.68	87.5%
36	Modernization and rehabilitation of the technologies and processes to reduce energy consumption in the pharmaceutical and sanitary industries	4.00 ± 0.81	81.2%
37	Using modern methods to predict the outbreak of climate change-sensitive diseases	4.43 ± 0.51	100%
	**Financial affairs**	**Mean**	**Consensus (%)**
38	Encouraging the authorities to allocate the funds in order to reduce the health effects of climate change in the country’s annual budget	4.18 ± 1.10	81.2%
39	Funding for topics related to the health and climate change in the Ministry of Health	4.31 ± 1.01	93.8%
40	Obtaining macroeconomic support at the High Council of Economics on climate change and health issue	4.12 ± 0.80	75%
41	Providing the insurance coverage for the health centers to enhance the resilience in extreme climate events	3.06 ± 0.92	75%
	**Assessment, evaluation and monitoring**	**Mean**	**Consensus (%)**
42	Continuous and systematic monitoring of the processes and measures taken to deal with the health effects of climate change	4.00 ± 1.15	75%
43	Vulnerability assessment of different population groups	4.06 ± 1.06	75%
44	Evaluating the effectiveness of adaptation and mitigation measures	4.06 ± 1.18	81.2%
45	Monitoring health information to provide early warning about the health effects of climate change	4.56 ± 0.62	93.8%
46	Cooperation with the country’s meteorological organization in monitoring of climatic variables (temperature and precipitation), amount of pollutants, and suspended particulate matter	4.00 ± 1.09	75%
47	Evaluating the effectiveness of adaptation interventions	4.50 ± 0.63	93.8%
48	Using health data in simulation and prediction models of disease burden	4.43 ± 0.72	87.5%
49	Monitoring of diseases sensitive to climate change through the national system in order to register the diseases	4.56 ± 0.51	100%
50	Monitoring of the climatic abnormalities including pollutants, dust, heat waves, cold waves, and providing related health plans	4.18 ± 0.98	87.5%
51	Diagnosing and rapid treatment of climate change-related diseases	4.31 ± 0.60	93.8%
52	Developing early warning and monitoring systems for heat and cold stresses as well as climate hazards	3.93 ± 1.34	75%
53	Assessing the deaths and injuries caused by the extreme climate events	4.06 ± 0.80	81.2%
	**Education and research**	**Mean**	**Consensus (%)**
54	Providing scientific evidence related to health and climatic variables in order to implement the health-related interventions	4.31 ± 0.60	93.8%
55	Increasing the knowledge and skills of health system staff about direct and indirect health effects of climate change	4.75 ± 0.44	100%
56	Collaboration with other educational institutions to strengthen the research and transfer of scientific knowledge related to health and climate change	4.37 ± 0.61	93.8%
57	Holding the conferences and workshops in relation to adaption to the effects of climate change	4.25 ± 0.68	87.5%
58	Collaboration with the public and private institutions to promote the education on health-related effects of climate change	4.25 ± 0.68	87.5%
59	Integrating the research findings into health policy-making to reduce the health risks	4.50 ± 0.63	93.8%
60	Developing human and research resources in the studies related to the health and climate change	4.18 ± 0.98	75%
61	Developing the educational headings on increasing the adaptation, reducing the vulnerability, and mitigation for students and university graduates	4.37 ± 1.02	93.8%
62	Collaboration with the international research centers to apply the updating science on the health effects of climate change	4.43 ± 0.81	93.8%
63	Increasing the possibility of participation and presence of health researchers in academic communities associated with climate change	3.81 ± 0.75	75%
64	Using the modern science and knowledge to enhance the individual and community adaptation	4.06 ± 1.06	81.2%
65	Performing the applied research to create individual and community adaptation in order to deal with the health effects of climate change	4.18 ± 0.83	75%
	**Reporting and sharing information**	**Mean**	**Consensus (%)**
66	Sharing the information and knowledge between the academic centers, the Ministry of Health and other relevant organizations to prevent the parallel works	4.12 ± 1.02	87.5%
67	Active participation of the health sector in climate change conventions and related scientific assemblies	4.37 ± 0.80	93.8%
68	Using all the available information and information synergies between the partner organizations	4.18 ± 0.83	75%
69	Applying the experiences and lessons learned from international conventions by the health system	4.50 ± 0.63	93.8%
70	Providing continuous and periodic reports on health system achievements and interventions for controlling the health effects caused by the climate change	4.50 ± 0.63	93.8%
71	Holding annual and joint reviews meetings, and generalization of successful experiences and lessons learned from meetings of national groups and international agencies by the Department of Environment	4.06 ± 0.92	75%
	**Public education**	**Mean**	**Consensus (%)**
72	Cultivating and raising the public and individual awareness about the health effects of climate changes	4.56 ± 0.51	100%
73	Cooperation with the medias to develop the educational programs for health warning related to climate change by the Ministry of Health	4.68 ± 0.47	100%
74	Providing the education in schools and higher education institutions about climate change and related health effects	4.31 ± 0.60	93.8%
75	Conducting the public and specialized conferences on health and climate change	4.00 ± 0.89	75%
76	Providing the educational packages including videos and pamphlets by the Ministry of Health on how to use personal protective equipment and adapt to health-related consequences of climate change	4.25 ± 0.77	81.2%
77	Increasing the public awareness about self-care in coping with the climatic disasters	4.62 ± 0.71	87.5%
78	Publishing the results of the research associated with climate change for the public	4.25 ± 0.68	87.5%
79	Enhancing the ability and readiness of the community to counteract the adverse health effects of climate change and extreme climate events	4.18 ± 0.75	81.2%

As mentioned before, the Paris Agreement on Climate Change has two main pillars “Mitigation” and “adaptation”. Accordingly, some recommendations have been made in the text of the agreement that can be used generally for all sectors, including energy, agriculture and industry. All of these measures can ultimately affect human health. The health sector not only could be affected by the consequences of climate change, but actually it could be an influential sector on climate change and greenhouse gas emission. Table 3 lists all components that have been generalized to the health sector. In the following, Table 4 describes the strategies and action plans proposed by health experts for mitigation and adaptation measures to combat the health consequences of climate change in more detail. The components listed in the table have a high consensus among experts.

## Discussion

This study was conducted to investigate the role of the health system in relation to two major pillars of the Paris Agreement, namely adaptation and mitigation. The health system is not the custodian for the mitigation measures; hence, its contribution to mitigation is very low compared to other sectors such as energy, industry, environment, and agriculture. Nevertheless, through its advocacy role, the health system can take advantage of the mitigation measures. Our research showed that the health system in Iran can play a role in mitigation measures, i.e. through active participation of the MoHME in the formulation of strategies and programs, as well as taking measures with respect to the mitigation, establishment of green hospitals, increasing the efficiency of energy carriers in healthcare facilities, using renewable energy, reducing waste production in health centers and all health-related manufacturing units in the areas of cosmetics, health, and pharmaceuticals.

Although limited data exists on tracking the health sector’s carbon footprint, The health sector has been estimated to be responsible for about 3–8% of all GHG emissions in some industrialized countries [36], while the figure is expected to be lower in the less-industrialized countries. Some studies indicate that for the period of 2003–2013, the US health sector has also been estimated to be responsible for a significant contribution to air pollution and its effects including acid rain (12%), greenhouse gas emissions (10%), air pollutants (9%), ozone depletion of the stratosphere (1%), and carcinogenic and non-carcinogenic toxins (1–2%). A previous study stated that, the concerted efforts to improve the environmental performance of health care centers by reducing the waste and energy production, and the burden of pollution are effective [37]. There is very little information on GHG emissions from health systems in low and middle-income countries, which highlights the need for more research in this area [38]. Carbon reduction measures are not only excellent opportunities for financial management in the health sector, but also might help improve the healthcare resilient infrastructures [38]. Previous studies showed that these measures including access to green and renewable energy [39, 40], green health centers [41], using low-consumption heating and cooling and ventilation systems, reducing distance to health facilities for patients in order to reduce the consumption of transportation energy, and management of procurements, materials, and wastes can contribute to lowering GHG emission [38].

Adaptation is the second main strategy that set out in Paris Agreement on climate change to tackle the effects of climate change. Considering the inevitability of the climate change phenomenon, adapting to it is crucial now more than ever. Our findings identified capacity building as one of the obvious examples of adaptation in the health system of Iran. Accordingly, capacity building is important in the form of resiliency strategies and adaptation plans for the health and climate change issues, increasing the capacity of healthcare infrastructures to cover full services in extreme climate events, developing health insurance to improve the adaptation; formulation of preparedness plans for health sector at the national, local, regional, and international levels, and integrating a comprehensive risk assessment plan into the health service delivery system to reduce mortality and injury in extreme climate events. It also helps identify the vulnerable groups of the health system in order to increase the capacity and provide the services in vulnerable geographical locations.

The use of indigenous and local capacities to create the adaptation is a strategic decision. In the same vein, the participation of local public and private institutions aimed at adapting to the climate change and inter-departmental and international partnership in terms of health adaptation policies are among the important issues supporting other organizations to achieve healthcare-related goals. In 2015, WHO reported the health and climate policies, human capital development, information infrastructure, and development of health-related services to strengthen the resiliency and increase the capacity of health systems in a variety of climatic conditions as necessary [42].

With regard to the technologies, the use of new technologies in the area of consumption management plays a significant role, especially, in relation to reduction of waste production and processes related to the enhancement of energy efficiency. The use of these technologies to increase the adaptation can play an effective role in monitoring, outbreak prediction, prevention, and control of diseases that are sensitive to climate change. A study investigated the roles of telemedicine and health information technology in reducing the carbon footprint in the health sector [36, 38]. Some scholars introduce eHealth as an adaptive strategy to reduce social vulnerability to climate change and a promising technology for mitigation in the health sector [43].

Financial issues related to the health sector and climate change are important and challenging mitigation initiatives and their costs should be calculated for budgeting the MoHME and other relevant organizations.

Evaluation and monitoring health information has always been considered as one of the important processes in the health system management. Our findings revealed the measures required in this regard including the vulnerability assessment of the affected population; continuous and systematic monitoring and evaluation of the effectiveness of adaptive interventions; use of Early Warning Systems to warn against the health effects of climate change and climatic anomalies; collecting health data related to these effects and using them in simulation models.

There are some evidence-based evaluation methods available for policymakers to increase the resiliency against the health effects of climate change including the assessment of burden of diseases related to climate change, evaluation of the adaptation interventions, and estimation of the potential health effects, and using future climate change scenarios [44].

In terms of reporting and information sharing, complementary to the efforts by the National Climate Change Workgroup at the DoE, which acts as the national authority of the climate change, the health system can also play an important role in formulating health-related appendices to all national and international reports. Relevant stakeholders are expected to contribute to the systematic and periodic reporting of health system achievements and interventions to control the health effects of climate change, and share the reports of relevant interventions with the international organizations. The MoHME needs to join the DoE in learning the lessons from international conventions and related scientific assemblies, while providing feedback to them and their participation in such meetings will be crucial.

The MoHME in Iran is responsible for both provision of public health services as well as enhancing health standards through an educational approach. Our study showed that production of scientific evidence related to climate variables for health interventions, as well as increasing the knowledge and skills of health system staff about the direct and indirect effects of climate change on the health system, are two essential function of Iran’s health system. We advocate a greater collaboration between the health system and other educational institutions in Iran, i.e. the Ministry of Science, Research, and Technology, and the Ministry of Education to strengthen knowledge transfer related to health and climate change. This could be conducted through scholarly meetings, conducting applied research, research collaborations with national and international organizations, and strengthening the educational curricula. Education can be discussed in both specialized and general areas. In particular, the MoHME needs to collaborate with, through the ministry of education, schools for fostering the use of modern knowledge to increase pupils’ awareness about the health effects of climate change. This may create in turn higher adaptation in the form of self-care programs and through educational packages such as films and educational pamphlets, which are important means to understand the relationship between the environment and health. This can also help the next generation create a healthier and more secure future [45], through integrating the subject of climate change into medical curricula via the best innovations made in medicine [46].

Our findings are also consistent with those presented in the WHO report of 2015, which offered an operational framework, comprising of 10 key functions to create a resilient health system against climate change. These include health governance and policy, human resources for health, supplying human resources, integrated risk monitoring and early warning systems, vulnerability, capacity and adaptation assessment, essential products and technologies, risk monitoring and early warning system, climate-informed health programs, emergency preparedness and disaster risk management, and management of the environmental determinants of health, research, and financing [47]. In addition, in 2014, the US Center for Disease Control and Prevention (CDC) put forward operational priorities for protecting health against climate change. These priorities include tracking the environmental information and the prevalence of the diseases related to the climate change, modeling and predicting the health effects of the climate change, identifying the locations and populations at risk, developing and implementing the preparedness and response plans for extreme climate events, providing counseling services to public and local health sectors, and providing the workforce responding to the health threats of climate change [48].

Our study was conducted based on the Paris Agreement on climate change, which is the most up-to-date global policy on adaptation and mitigations measures. Since this agreement observes the “right to health” in its approach, and because of WHO’s emphasis on the development and implementation of adaption strategies, our findings provided an evidence-based basis for future policy-making in the health system of Iran, and perhaps similar middle-income countries, towards a meaningful adaptation to climate change.

## Conclusion

The growing trend of health risks related to climate changes highlights the need for developing tailored programs to adapt and mitigate its health effects. Evidence-based policy-making is a fundamental tool to formulate effective programs in this regard. Considering the main elements of the Paris Agreement (as the most up-to-date global policy on climate change) regarding the mitigation and better adaptation against the negative effects of climate change, translation and customization of Paris Agreement on climate change is necessary to direct the Iranian MoHME to make appropriate policies within the political context of Iran. This approach can pave the way for further steps towards capacity building and enhancement of resilience of the health system, adaptation interventions, evaluation, identification of barriers and facilitators for mitigation and adaptation to the adverse health effects caused by the climate change. Our findings can be used to support targeted dissemination of required components for influencing the implementation and realization of the domesticated model of Paris Agreement on climate change in Iran and perhaps beyond. As Iran has signed the Paris Agreement, and along its long pathway to achieve sustainable development goals, policymakers need to design customized plans and policies to decrease the negative effects of the climate change.

## Supplementary information

**Additional file 1: Supplementary file**. Questionnaire. Researcher-made questionnaire: First round of Delphi. As a part of research at Tehran University of Medical Sciences, we conducting a survey that extracts the components of functions and policymaking to provide an evidence-based framework for health policy-making towards reducing greenhouse gases emissions and adapting to the health effects of climate change in Iran.

## Data Availability

Not applicable.

## Abbreviations

SDGs: Sustainable Development Goals
SDH: Social Determinants of Health
WHO: World Health Organization
UNFCCC: United Nations Framework Convention on Climate Change
WHA: World Health Assembly
HDI: Human Development Index
PHC: Primary healthcare network
UHC: Universal Health Coverage
DoE: Department of Environment
IER: Institute for Environmental Research
NCDs: Non- communicable diseases
CVDs: Cardiovascular diseases
CCHF: Crimean-Congo Hemorrhagic Fever
MoHME: Ministry of Health and Medical Education
GHGs: Greenhouse Gases
EWS: Early Warning Systems
CDC: Center for Disease Control and Prevention
