# Supplementary material for: Health system plan for implementation of Paris agreement on climate change (COP 21): a qualitative study in Iran
Source: BMC Public Health. 2020 Sep 11;20:1388. doi: 10.1186/s12889-020-09503-w (PMC7488526; doi:10.1186/s12889-020-09503-w)
Supplement: Supplementary file 1 — Additional file 1: Supplementary file. Questionnaire. Researcher-made questionnaire: First round of Delphi. As a part of research at Tehran University of Medical Sciences, we conducting a survey that extracts the components of functions and policymaking to provide an evidence-based framework for health policy-making towards reducing greenhouse gases emissions and adapting to the health effects of climate change in Iran. [file 12889_2020_9503_MOESM1_ESM.docx]

**Researcher-made questionnaire: First round of Delphi**

As a part of research at Tehran University of Medical Sciences, we conducting a survey that extracts the components of functions and policymaking to provide an evidence-based framework for health policy-making towards reducing greenhouse gases emissions and adapting to the health effects of climate change in Iran. I will appreciate if you could complete the following table.

**Name:**

**Affiliation:**

| **Strongly Agree (5)** | **Agree (4)** | **Neutral (3)** | **Disagree (2)** | **Strongly Disagree (1)** |
| --- | --- | --- | --- | --- |

|  | **Components of Paris Agreement on Climate Change** | **Relevance to health systems performance** | | | | |
| --- | --- | --- | --- | --- | --- | --- |
|  | **Mitigation** | **1** | **2** | **3** | **4** | **5** |
| 1 | Holding the increase in the global average temperature to well below 2 °C above pre-industrial levels |  |  |  |  |  |
| 2 | Increasing the ability to adapt to the adverse impacts of climate change and foster climate resilience and low greenhouse gas emissions development, in a manner that does not threaten food production; |  |  |  |  |  |
| 3 | Rapid reduction of greenhouse gas emissions in accordance with best available science |  |  |  |  |  |
| 4 | Developing strategies, plans and implementing the mitigation measures |  |  |  |  |  |
| 5 | Reducing emissions from deforestation and forest degradation |  |  |  |  |  |
| 6 | Creating a balance between anthropogenic emissions by sources and removals by sinks of greenhouse gases |  |  |  |  |  |
| **Adaptation** | | **1** | **2** | **3** | **4** | **5** |
| 7 | Increasing the ability and capacity on local, national, regional, and international levels to adapt to the adverse effects of climate change |  |  |  |  |  |
| 8 | Creating the adaptation based on the current, past, indigenous, and local knowledge |  |  |  |  |  |
| 9 | Integrating the adaptation in the policies, social, economic, and environmental measures |  |  |  |  |  |
| 10 | Creating country-oriented, gender-oriented, and participatory adaptation with regard to sensitive groups, communities, and ecosystems |  |  |  |  |  |
| 11 | Adjustment, implementation and monitoring of National Adaptation Strategies and Plan of Actions |  |  |  |  |  |
| **Capacity building** | | **1** | **2** | **3** | **4** | **5** |
| 12 | Capacity building for mitigation measures based on the local, national, and regional needs |  |  |  |  |  |
| 13 | Capacity building for adaptation measures based on local, national, and regional needs |  |  |  |  |  |
| 14 | Improving the resiliency to climate change |  |  |  |  |  |
| 15 | Reducing the vulnerability to climate change |  |  |  |  |  |
| **Advocacy** | | **1** | **2** | **3** | **4** | **5** |
| 16 | Promoting voluntary cooperation in climate change measures |  |  |  |  |  |
| 17 | Encouraging and facilitating the participation of public and private institutions in climate change measures |  |  |  |  |  |
| **Technology development** | | **1** | **2** | **3** | **4** | **5** |
| 18 | Developing and transferring the technology to to reduce greenhouse gas emissions |  |  |  |  |  |
| 19 | Developing and transferring the technologies to increase the adaptation |  |  |  |  |  |
| **Financial affairs** | | **1** | **2** | **3** | **4** | **5** |
| 20 | Providing financial support for developing and transferring the technologies at different stages of the technology life cycle |  |  |  |  |  |
| 21 | Reducing the costs of adaptation measures through increasing mitigation measures |  |  |  |  |  |
| 22 | Providing financial resources to reduce greenhouse gas emissions |  |  |  |  |  |
| 23 | Providing financial resources to increase the adaptation and resilience to climate change |  |  |  |  |  |
| **Assessment, Evaluation and Monitoring** | | **1** | **2** | **3** | **4** | **5** |
| 24 | Assessing the adequacy and effectiveness of mitigation measures |  |  |  |  |  |
| 25 | Assessing the adequacy and effectiveness of adaptation measures |  |  |  |  |  |
| 26 | A systematic monitoring of climate system and early warning systems |  |  |  |  |  |
| 27 | Assessing the effects of climate change |  |  |  |  |  |
| 28 | Assessing the vulnerability to climate change |  |  |  |  |  |
| **Extreme weather events** | | **1** | **2** | **3** | **4** | **5** |
| 29 | Minimizing the damage caused by the effects of extreme climate events |  |  |  |  |  |
| 30 | Developing early warning systems for extreme climate events |  |  |  |  |  |
| 31 | Being prepared for extreme climate events |  |  |  |  |  |
| 32 | Assessing the damages caused by the extreme climate events |  |  |  |  |  |
| 33 | Risk management of extreme climate events |  |  |  |  |  |
| 34 | Comprehensive risk assessment of extreme climate events |  |  |  |  |  |
| 35 | Providing risk insurance facilities, climate risk pooling, and other insurance solutions |  |  |  |  |  |
| **Education and research** | | **1** | **2** | **3** | **4** | **5** |
| 36 | Strengthening scientific knowledge about climate change and its effects |  |  |  |  |  |
| 37 | Promoting the education on climate change and its effects |  |  |  |  |  |
| 38 | Promoting the research on climate change and its effects |  |  |  |  |  |
| **Reporting and sharing information** | | **1** | **2** | **3** | **4** | **5** |
| 39 | Integrating and sharing of information, knowledge, appropriate actions, experiences, and lessons learned |  |  |  |  |  |
| 40 | Providing timely and accurate reporting of information |  |  |  |  |  |
| 41 | Using lessons learned from the International Climate Change Convention frequently and effectively |  |  |  |  |  |
| 42 | Providing regular reports on the progress of the policies, programs, and undertaken actions |  |  |  |  |  |
| **For public** | | **1** | **2** | **3** | **4** | **5** |
| 43 | Raising the public awareness on climate change and its effects |  |  |  |  |  |
| 44 | Raising the public awareness and transparency on mitigation measures |  |  |  |  |  |
| 45 | Raising the public awareness and transparency on increasing the adaptation and resiliency measures |  |  |  |  |  |
| 46 | Promoting the public participation on measures related to climate change |  |  |  |  |  |
| 47 | Promoting the public access to information on climate change and its effects |  |  |  |  |  |
